# Histoplasmosis-Induced Hemophagocytic Lymphohistiocytosis in an Adult Patient: A Case Report and Review of the Literature

**DOI:** 10.1155/2016/1358742

**Published:** 2016-10-18

**Authors:** Ramona Vesna Untanu, Syed Akbar, Stephen Graziano, Neerja Vajpayee

**Affiliations:** ^1^Department of Pathology, SUNY Upstate Medical University, Syracuse, NY 13210, USA; ^2^Department of Internal Medicine, SUNY Upstate Medical University, Syracuse, NY 13210, USA

## Abstract

Hemophagocytic lymphohistiocytosis (HLH) is an aggressive and life-threating immune dysregulation syndrome characterized by persistent activation of the mononuclear phagocytic system leading to uncontrolled systemic hyperinflammatory response. The proliferation and activation of histiocytes and lymphocytes lead to production of large amounts of cytokines, also called cytokine storm. Hematopoietic and lymphoid tissues are directly involved while other organs are damaged by circulating cytokines. Primary HLH is attributed to genetic defects of the immune system and secondary HLH is usually seen in adults secondary to malignancy, infection, or autoimmune diseases. Zoonotic diseases including fungal infections are an important cause of HLH. Secondary HLH can delay the recognition of the underlying zoonoses. We report the case of a 61-year-old female with history of rheumatoid arthritis with histoplasmosis associated hemophagocytic lymphohistiocytosis.

## 1. Introduction

Hemophagocytic lymphohistiocytosis (HLH) is a disorder of regulatory immunomodulatory pathways inciting phagocytosis of hematopoietic cells resulting in end-organ damage. The condition often results in a fatal outcome without prompt diagnosis and treatment [1]. The pathogenesis of HLH remains to be fully elucidated. A recent review of all animal models for primary and secondary HLH highlights the major role of interferon-gamma in the cytokine storm, supported by the efficacy of interferon-gamma blockers for primary HLH only [2]. Many of the secondary HLH cases may be due to an underlying genetic predisposition [1, 3, 4]. Hemophagocytosis is the histopathologic feature present in bone marrow, spleen, liver, or lymph nodes. HLH is most common in young children but can present at any age. HLH is classified in two categories: primary (familial) and secondary (acquired) [1, 3]. Primary HLH is attributed to genetic defects of T and NK cells cytotoxic function (PRF1, UNC13D, STX11, RAB27A, LYST, AP3B1, and SH2D1A) and is seen mostly in the pediatric age group [3]. Familial HLH is an autosomal recessive or X-linked disease and is usually triggered by viral infection. Secondary HLH is usually seen in adults where malignancy (usually hematologic), infection, or autoimmune diseases cause an acquired defect in cytotoxic function of lymphocytes or NK cells. Unlike pediatric cases, adult HLH is frequently due to underlying infection, malignancy, or autoimmune disease [4–6]. A review of HLH in rheumatic patients highlights the importance of recognizing HLH in this clinical context as underdiagnoses can affect prognosis [7]. Zoonotic diseases are an important cause of HLH. Secondary HLH can delay the recognition of the underlying zoonoses and contribute to the poor outcome. Among zoonotic fungi, most secondary HLH cases are reported in AIDS patients with histoplasmosis [8].

## 2. Case Presentation

**Figure 1:**
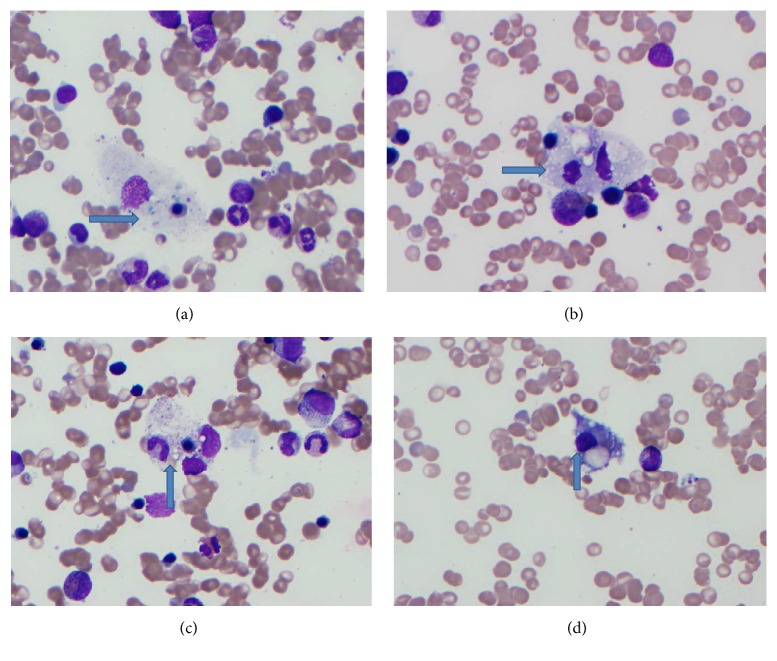
(a)–(d) Wright Giemsa stained bone marrow aspirate smear with increased number of histiocytes depicting hemophagocytosis (engulfed white and red blood cell precursors).

**Figure 2:**
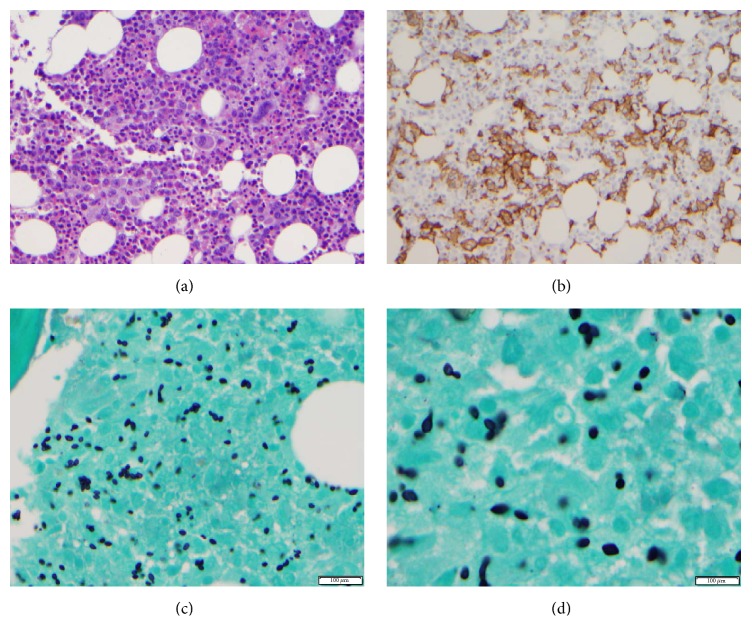
(a) Bone marrow biopsy with increased number of histiocytes, focally present in loose aggregates, hematoxylin and eosin stain, 40x. (b) CD163 staining on bone marrow biopsy highlighting histiocytes. (c)-(d) GMS (Gomori-Methenamine Silver) staining on bone marrow biopsy. Numerous budding fungal organisms with morphology consistent with* Histoplasma* are present.

We report the case of a 61-year-old patient with history of rheumatoid arthritis treated with remicade and methotrexate who presented with fever, malaise, anorexia, weight loss, cough, and jaundice for a month. She also had a diffuse morbilliform rash which on biopsy revealed superficial perivascular dermatitis without any specific immunoglobulin and/or complement deposition. Physical exam revealed splenomegaly. Laboratory tests revealed hypoalbuminemia with transaminitis, elevated alkaline phosphatase (179 U/L), hyperbilirubinemia (1.7 mg/dL), hypertriglyceridemia (453 mg/dL), elevated IL-2 (13321 U/mL), and ferritin (5870 ng/dL which increased to 20,644 ng/dL during the course of her hospital stay). CBC showed normocytic anemia with a hemoglobin of 8.1 g/dL, and thrombocytopenia with a platelet count of 120 K/uL. WBC was normal at 10.1 K/uL with mild left shift in the granulocytic series. Initial infectious workup was negative. Bone marrow study was performed to further investigate for cause of cytopenias and persistent fever. Aspirate smears showed numerous macrophages with evidence of hemophagocytosis (Figures 1(a)–1(d)). The biopsy was slightly hypercellular, showing low normal myeloid to erythroid ratio, megakaryocytes were slightly increased in number, and increased proportion of histiocytes, focally also forming loose aggregates, were noted. Histiocytes had foamy cytoplasm and in a few cells intracytoplasmic cellular debris was also identified. Well defined granulomas or areas of necrosis were not seen (Figures 2(a) and 2(b)). Staining for fungal organisms (GMS) revealed budding yeast with morphology consistent with* Histoplasma*. Further CD163 immunostain highlighted increased proportion of histiocytes in the bone marrow biopsy. Blood cultures were positive for* Histoplasma capsulatum*.

As the patient met the diagnostic criteria for HLH, she was started on HLH-94 protocol with Decadron (10 mg/m^2^) and etoposide (150 mg/m^2^) and showed some improvement. She had received two doses of etoposide before the results of bone marrow biopsy and blood cultures came out. She subsequently received IV amphotericin B for nine days and was then switched to oral itraconazole. Patient was discharged after improvement of her symptoms. Prior to discharge the serum ferritin and liver enzymes were trending down and repeat fungal blood cultures were negative. One-month follow-up showed normal CBC and liver enzymes and patient was asymptomatic.

## 3. Discussion

HLH can be difficult to recognize due to the variety of etiologic factors, nonspecific clinical symptoms, and laboratory data. Abnormal liver function, cytopenia, and very high ferritin level are most useful in differentiating HLH from other entities. HLH can simulate infections/sepsis, hepatitis, multiple organ dysfunction syndrome, encephalitis, autoimmune lymphoproliferative syndrome, thrombotic thrombocytopenic purpura, or fever of unknown origin. Infection has been shown in a study to be the major trigger of HLH in adults treated with biological therapies [9].

Patients present acutely with fever and multiple organ involvement including hepatomegaly, splenomegaly, lymphadenopathy, cutaneous manifestation, neurologic symptoms, pulmonary symptoms, renal dysfunction, or hypotension [3, 10]. Dermatologic findings include generalized rash, petechiae, and purpura. Studies report the median age in adults of 51–53 years (range, 18–82) with a slight male predominance (56.2–63%) [5, 6]. Laboratory features of HLH include cytopenia (especially anemia and thrombocytopenia), increased ferritin level (usually very high), abnormal liver function tests, increased triglycerides, and coagulation abnormalities (PT, PTT, fibrinogen, and D-dimer) due to liver dysfunction or disseminated intravascular coagulation. Liver function tests can show elevated liver enzymes (AST, ALT, and GGT), LDH, and bilirubin or low albumin level. Fever, cytopenia, and elevated ferritin were observed in >85% of cases in a study by Otrock and Eby [5]. Elevated ferritin is less specific for adult HLH and it can be due to other inflammatory conditions [4]. Blood cultures should be performed to determine an infectious cause. Imaging studies might be helpful if an underlying malignancy is suspected. CSF is abnormal in 50% of cases with pleocytosis, elevated protein, or hemophagocytosis [3, 10]. Diagnostic criteria for HLH are listed in the following. 

*Diagnostic Criteria for HLH Used in the HLH-2004 Trial. *The diagnosis of HLH may be established by the following:

- Molecular diagnosis consistent with HLH is as follows: pathologic mutations of PRF1, UNC13D, Munc18-2, Rab27a, STX11, SH2D1A, or BIRC4.
- Five of the 8 criteria listed below are fulfilled:
  1. Fever > or equal to 38.5°C
  2. Splenomegaly
  3. Cytopenias (affecting at least 2 of 3 lineages in the peripheral blood)
    1. Hemoglobin < 9 g/dL (in infants < 4 weeks: hemoglobin < 10 g/dL)
    2. Platelets < 100 × 10^3^/mL
    3. Neutrophils < 1 × 10^3^/mL
  4. Hypertriglyceridemia (fasting, >265 mg/dL) and/or hypofibrinogenemia (<150 mg/dL)
  5. Hemophagocytosis in bone marrow or spleen or lymph nodes or liver
  6. Low or absent NK cell activity
  7. Ferritin > 500 ng/mL (whereas the HLH-2004 protocol uses ferritin 500 ng/mL, we generally view ferritin 3000 ng/mL as concerning for HLH and ferritin 10,000 as highly suspicious in pediatric patients.)
  8. Elevated soluble CD25 (soluble IL-2 receptor alpha) (elevations above age-adjusted, laboratory-specific normal levels (defined as 2 SD from the mean) appear more meaningful than the original designation of “2400 U/mL,” because of variations between laboratories.)

The diagnostic criteria have been adapted from Henter et al. [10]. Quantitative flow cytometric findings in HLH, regardless of etiology, include decreased myeloid fraction with left shifted myelopoiesis and increased lymphoid cells with abundant cytotoxic T cells [11]. Most EBV related HLH cases show expanded atypical CD8 T cells population with variable loss of expression of CD5, CD7, or CD3 and abnormal myeloid immunophenotype with lack of CD10 and expression of HLA-DR [11]. Flow findings in HLH are nonspecific but different than uninvolved marrow and should not be overinterpreted. The histopathologic hallmark of HLH is hemophagocytosis. Hemophagocytosis is defined by macrophage engulfment of red blood cells, white blood cells, platelets, and their precursors. It can be seen in the bone marrow, lymph nodes, spleen, and liver. Besides HLH, hemophagocytosis is also associated with infection, inflammation, marrow hyperplasia, ineffective hematopoiesis, blood transfusion, chemotherapy, major surgery, and malignancies. The involved hematopoietic tissues can sometimes show bland lymphohistiocytic infiltrates without hemophagocytosis [10]. In such cases, CD163 or CD68 immunostains can help highlight the histiocytes. Liver biopsy can show a picture of chronic hepatitis with periportal lymphocytic infiltrate [10]. Hemophagocytosis is not specific or necessary for a diagnosis of HLH if other criteria are met. A study of 80 bone marrow cases with hemophagocytosis evaluating differences between HLH and non-HLH cases concludes that hemophagocytosis should always be documented as it may be the only clue of infection [12]. Pancytopenia and higher grade of hemophagocytosis may differentiate HLH versus non-HLH cases [12]. In contrast, another study suggests that hemophagocytosis, even at high degree lacks specificity for HLH and shows poor correlation between hemophagocytosis and HLH probability [13]. Bone marrow assessment remains valuable as it can rule out other diseases related or not to HLH. A study evaluating the role of the initial marrow aspirate in HLH diagnosis concludes that hemophagocytic cells can be few and variable and a negative bone marrow does not exclude HLH and should not delay treatment [14]. Special stains for identification of microorganism might be helpful in cases suspicious for infection (fungal, mycobacterial).

HLH-2004 chemoimmunotherapy includes etoposide, dexamethasone, cyclosporine A upfront, and, in selected patients, intrathecal therapy with methotrexate and corticosteroids [10]. Subsequent hematopoietic stem cell transplantation (HSCT) is recommended for patients with familial disease or molecular diagnosis, and patients with severe and persistent, or reactivated, disease [10].

Histoplasmosis-induced HLH is a rare entity and there is insufficient data to establish the best treatment protocol and outcome [15]. Survival of histoplasmosis-induced HLH patients is very low and most are HIV/AIDS cases [15]. A reported case of HLH in a patient with HIV, disseminated histoplasmosis,* Mycobacterium avium* complex, and* Escherichia coli* bacteremia showed a good outcome based on treatment of underlying infections and without immunochemotherapy [16].* Histoplasma duboisii*, a variant of* Histoplasma capsulatum* that causes “African histoplasmosis,” can be resistant to itraconazole and rarely even to intravenous antifungal therapy and may progress to secondary HLH and death.

Adult studies report a poor median overall survival of 7.67–4 months with a markedly worse survival for malignancy-associated HLH compared to HLH without malignancy [5, 6]. Nikiforow and Berliner showed a very poor prognosis for malignancy-associated HLH [4]. High serum beta2 microglobulin and lymphoma associated hemophagocytic syndrome are associated with markedly poorer overall survival in HLH patients [17]. Beta2 microglobulin is a strong and independent prognostic factor for overall survival in lymphoma associated hemophagocytic syndrome [17]. Remission in secondary HLH following treatment of underlying disease has been reported in a Brazilian series [18]. For primary HLH, immunochemotherapy can lead to remission but bone marrow transplant is needed for cure [18].

## 4. Conclusion/Summary

HLH is a severe, potentially fatal disease that requires early recognition and assessment of secondary causes. Urgent treatment is required and therapeutic modalities may differ according to the underlying disease. A better understanding of adult HLH pathogenesis is needed to personalize treatment protocols that are based on pediatric patients. Further studies are necessary to develop more effective therapies, improve survival, and validate current diagnostic criteria.
